# Burns and biofilms: priority pathogens and in vivo models

**DOI:** 10.1038/s41522-021-00243-2

**Published:** 2021-09-09

**Authors:** Evgenia Maslova, Lara Eisaiankhongi, Folke Sjöberg, Ronan R. McCarthy

**Affiliations:** 1 Division of Biosciences, Department of Life Sciences, College of Health and Life Sciences, Brunel University London, Uxbridge, UK; 2 Department of Hand and Plastic Surgery, The Burn Centre, Linköping University, Linköping, Sweden; 3 Department of Clinical and Experimental Medicine, Faculty of Health Sciences, Linköping University, Linköping, Sweden

**Keywords:** Clinical microbiology, Pathogens, Biofilms

## Abstract

Burn wounds can create significant damage to human skin, compromising one of the key barriers to infection. The leading cause of death among burn wound patients is infection. Even in the patients that survive, infections can be notoriously difficult to treat and can cause lasting damage, with delayed healing and prolonged hospital stays. Biofilm formation in the burn wound site is a major contributing factor to the failure of burn treatment regimens and mortality as a result of burn wound infection. Bacteria forming a biofilm or a bacterial community encased in a polysaccharide matrix are more resistant to disinfection, the rigors of the host immune system, and critically, more tolerant to antibiotics. Burn wound-associated biofilms are also thought to act as a launchpad for bacteria to establish deeper, systemic infection and ultimately bacteremia and sepsis. In this review, we discuss some of the leading burn wound pathogens and outline how they regulate biofilm formation in the burn wound microenvironment. We also discuss the new and emerging models that are available to study burn wound biofilm formation in vivo.

## Introduction

Wound infection is one of the main clinical complications associated with wound care. In burn patients, in particular, the leading cause of mortality is an infection, with 75% of all deaths from burns resulting from infection^1^. Burn wounds are a severely debilitating class of wound and can have a life-long impact on a patient’s health. Annually around 265,000 deaths worldwide are attributed to fire-related burns alone, with 90% of all burns taking place in developing countries where the patient mortality reaches 100% with burns covering >40% of total body surface area. Within the UK the NHS manages ~90,000 burn-related hospital admissions, while globally millions of people suffer from burn-related injuries each year^2–4^. Wound care and management place an enormous burden on global health care systems with the UK’s National Health Service (NHS) estimated to spend ~5.3 billion pounds annually on wound management^3^.

Burn injury is a major challenge for the patient’s immune system, leaving them immunocompromised and vulnerable to pathogens such as nosocomial bacterial infections and multidrug-resistant pathogens^5,6^. The wound severity and prognosis depend on various factors, such as the surface area of the burn, the degree of the burn, the patient’s medical history, and their age. A superficial (first degree) burn spreads only to the epidermis. It is known to heal well, quickly, and without scarring^7^. Second-degree burns, or partial-thickness burns, involve the deeper layers of the epidermis and dermis and are slower to heal^8^. Third-degree (full thickness) burns destroy the epidermal and dermal layers and can involve damage to underlying tissue and bone^9^. Each of these different levels of burn wound represents a different threat in terms of bacterial infection as the deeper the burn, the higher the probability the pathogen can penetrate the circulatory system and cause bacteremia and sepsis. The burn wound itself is a complex microenvironment predominated with biological fluids known as burn wound exudates (BWE). The metabolic and cellular profile of these BWE creates a niche environment where certain pathogens with a high metabolic versatility can proliferate successfully^10^. Opportunistic pathogens such as *Pseudomonas aeruginosa, Acinetobacter baumannii*, and *Staphylococcus aureus*, are notorious for colonizing burn wounds. These pathogens in particular are commonly found in the hospital environment and have caused outbreaks in burn units globally^11–13^. The ability of these pathogens to form biofilms is a major contributor to their pathogenic success and considerably complicates burn wound management. It also negatively impacts the survival rate among burn victims^14,15^.

### Biofilms in wounds

**Fig. 1:**
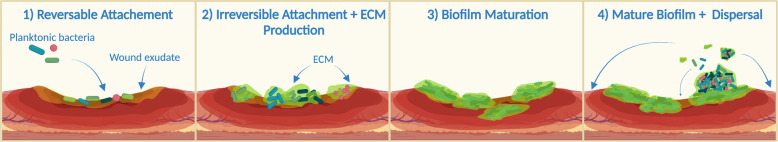
The proposed stages of biofilm bacterial infection development in the burn wound. (1) Burn wounds typically contain BWE which facilitates the initial inoculation and reversible attachment by planktonic pathogens. (2) Bacteria begin to produce ECM and form microcolonies during the process of irreversible attachment (3) During the maturation stage, the biofilm grows in size and structural complexity. (4) The mature biofilm enters the dispersal stage, releasing the planktonic cells from the ECM which can then colonize new sites within the wound. Created with BioRender.com.

A biofilm is an organized community of bacteria that have attached to a surface (biotic or abiotic) or to each other (aggregates). The formation of a biofilm begins with the reversible attachment of cells (Fig. 1). Cells then transition to the irreversible attachment stage and begin to produce the extracellular matrix (ECM). This ECM is primarily made up of water with its structural and functional integrity maintained by polysaccharides, proteins, lipids, and extracellular DNA (eDNA). As the cells begin to encase themselves in this matrix, the biofilm enters the maturation stage. Within a mature biofilm, bacteria are capable of distributing resources through channels and responding to internal and external environmental triggers. Finally, as a biofilm matures it can enter the dispersal phase, where planktonic cells and aggregates are released from the matrix, infecting new sites within the wound, and the cycle repeats^16,17^. Much of our understanding of how biofilm formation occurs within wounds is based on in vitro and ex vivo data and there is a need to further explore these life cycle stages in vivo. It is clear, however, that the biofilm mode of growth offers distinct advantages to bacteria in a wound, protecting them from the host immune system and antibiotics. Bacteria growing in a biofilm are thought to be between 10 and 1000 times more tolerant to antibiotics than their planktonic counterparts^18^. Consequently, bacteria in biofilms are notoriously difficult to eradicate from the wound site.

It is now well established that biofilm formation delays wound healing and can drive the development of chronic wounds. Chronic wounds are defined as wounds that take longer than 4 weeks to heal^19–21^. The process of wound healing is continuous and can be divided into four phases: (1) coagulation and hemostasis, (2) inflammation, (3) proliferation, and (4) remodeling^22^. Chronic wounds are thought to occur when wounds get stuck in a particular phase of this process. Biofilm formation is thought to trap wounds in the inflammation stage due to the inability of immune cells, which accumulate at the wound site, to eradicate the biofilm^23,24^. This accumulation of immune cells stimulates a persistent inflammatory state at the wound site, delaying re-epithelization and closure. A meta-analysis of the published data on chronic wounds suggests that almost 80% are associated with a biofilm^25^. The role of biofilm formation in acute wounds is less clear and is an area of much debate. Part of the reason for this debate is that practical diagnostics for the presence of biofilm within wounds are lacking^26^. Biofilms have, however, been identified in acute wounds but at a relatively low frequency (6%) and in vivo evidence indicates that biofilms can form in acute wound models from as early as 3 days post-trauma. Indeed, within in vivo models, it has been demonstrated that biofilm formation at the burn eschar can precede systemic infection^27,28^. This suggests that biofilms may have a role in acute infection progression also, acting as a launchpad for deeper tissue invasion leading to bacteremia and sepsis^27,29,30^. In this review, we will discuss what is currently known about the regulatory mechanisms controlling burn wound biofilm formation in three of the most prevalent burn wound pathogens *Pseudomonas aeruginosa*, *Acinetobacter baumannii,* and *Staphylococcus aureus*. We also discuss the new and emerging in vivo models that can be used to study wound-associated biofilms.

### *P. aeruginosa* biofilms in burn wound infection

*P. aeruginosa* is an opportunistic Gram-negative bacterium that can cause acute and chronic infections^31^. It is recognized as a critical cause of mortality and morbidity among burn patients with studies showing it can be responsible for as high as 77% of burn wound mortalities^32,33^. *P. aeruginosa* can cause cross-transmission and outbreaks within hospitals by circulating through contaminated areas leading to localized outbreaks in burn treatment centers^34^. The ability of *P. aeruginosa* to form biofilms is well known as a key virulence trait that is central to its pathogenic success. By forming a biofilm *P. aeruginosa* decreases antibiotic treatment efficacy resulting in more chronic infections and prolonged hospital stays^35,36^.

Burn wound biofilm formation in *P. aeruginosa* has been shown to be tightly controlled by a range of environmental and genetic factors. An important environmental factor influencing *P. aeruginosa* biofilm formation is iron availability. This is particularly true in burn wounds which are considered to be low iron environments. *P. aeruginosa* is adept at overcoming this limitation via a variety of iron acquisition systems which have been shown to be upregulated when *P. aeruginosa* is growing in human BWE^10^. The *P. aeruginosa* iron starvation sigma factor PvdS coordinates the production of the iron scavenging siderophore, pyoverdine, which can compete for iron in iron-limited environments^37^. In human BWE, the expression of the pyoverdine biosynthesis genes *pvdL* and *pvdS* was increased at early phases of *P. aeruginosa* infection, followed by reduced expression at later stages of growth^10,38^. Similarly, in burn wound tissue, *P. aeruginosa* has been shown to increase the expression of *pvdS*. In tandem, the expression of protease encoding genes *lasA* and *lasB* was also shown to increase, potentially to facilitate the destruction of the host tissue matrix, increasing the availability of free iron^28,39^. Mutants in genes involved in siderophore production have been shown to produce weak biofilms^40^ further highlighting the importance of iron acquisition systems to successful biofilm formation within a burn wound.

Exopolysaccharide production is recognized as a hallmark of burn wound biofilm formation. Alginate, Pel, and Psl are the three polysaccharides that can make up the *P. aeruginosa* EPS matrix, but the role of each in biofilm formation is strain-dependent. These polysaccharides facilitate immune evasion and antibiotic tolerance by protecting the cells within the biofilm from these external insults^41–43^. Alginate is composed of mannuronic and guluronic acids and functionally is involved in biofilm maturation. Its overproduction is linked to a hyper mucoidy phenotype commonly seen in cystic fibrosis lung isolates^44^. Schaber et al. used a mouse burn wound model and alginate-specific fluorescent antibodies to demonstrate that alginate is a component of burn wound biofilms^45^. Brandenburg et al*.* also reported that during *P. aeruginosa* full-thickness burn wound infection, the expression of key alginate biosynthesis genes (*algD, alg8*, and *algE*) was increased as early as 24 h post trauma^27,28^. Pel is an *N*-acetylglucosamine (GlcNAc)- and *N*-acetyl galactosamine (GalNAc)-rich polysaccharide while Psl is composed of repeating glucose, rhamnose, and mannose residues, respectively^46,47^. They play important roles in the initiation and maintenance of biofilm structure, by promoting attachment and facilitating the cell-to-cell interactions necessary to hold cells together. The loss of either has been shown to halt biofilm growth^48^. Of these two polysaccharides, Pel, in particular, has been implicated in burn wound biofilm formation. In full-thickness scald burn wound infection in rats, the expression of Pel polysaccharide biosynthesis genes (*pelB, pelC*, and *pelD*) was elevated^27,28^. No change in the expression of alginate or Pel biosynthesis genes was seen in BWEs^10^ which may suggest that expression is dependent on contact with the wound bed or may be due to strain-specific variations.

Biofilm formation in *P. aeruginosa* is predominantly regulated by the Quorum Sensing (QS) system^49^. *P. aeruginosa* has three QS systems, the LasI/LasR, RhlI/RhlR, and PQS System, each system is known to play a role in regulating biofilm formation^50^. In most Gram-negative bacteria, QS systems depend on acylated homoserine lactones (AHLs). In cases of high cell density, AHLs accumulate and act as ligands for cognate response regulators. LasI and RhlI in *P. aeruginosa* synthesize the two AHLs, *N*-3-oxododecanoyl homoserine lactone and *N*-butyryl-homoserine lactone, respectively^45^. Consequently, they regulate the activity of LasR and RhlR response regulators, which control the expression of crucial virulence factor genes and biofilm-associated genes^45^. Burned mice challenged with *P. aeruginosa* PAO1 lacking functional *lasRI* and *rhlRI* QS genes displayed a significantly reduced mortality rate compared to mice challenged with Wild Type PAO1, highlighting role of these QS systems in *P. aeruginosa* burn wound pathogenesis^49^. The LasRI and RhlRI QS systems have been shown to be active at early stages of growth in human BWE; however, Gonzalez et al*.* suggested that in wounds, QS is based on efficiency sensing instead of a density-dependent system^10^. Intriguingly, while the QS system can promote biofilm formation, strains lacking *lasI* and *rhlI* genes are still able to form biofilms in burn wounds, thus suggesting that cell-to-cell signaling in *P. aeruginosa* may not be essential for rapid biofilm development within the burn wound microenvironment^45^. However, the expression of all three QS systems is induced in a mixed-species rat biofilm model and *rhlI* was induced in the porcine mixed-species biofilm model^27,51^. Taken together, these studies demonstrate that QS can drive burn wound biofilm formation but may not be essential, however, the complete role of *P. aeruginosa* QS in biofilm production in burn wound infections is yet to be fully uncovered.

### *S. aureus* biofilm in burn wound infection

Some Gram-positive bacteria including *Staphylococci* are commonly found on healthy skin. This localized reservoir enables colonization of burn wounds within the first 48 h, making *S. aureus* one of the most common pathogens isolated from burn wounds^52,53^. The estimated cost for infections due to *S. aureus* is $450 million annually^54^. Like *P. aeruginosa*, *S. aureus* can adopt a planktonic or a biofilm mode of growth^55^. Biofilm formation acts as a significant pathogenicity factor for *Staphylococcus* spp. particularly in burn wounds^56^. The *agr* QS system in *S. aureus* uses peptides rather than AHLs as its autoinducing signal. It represses biofilm formation by decreasing the expression of cell-wall-associated adherence factors^54,55^. Upon exposure to burn serum, oxidative stress leads to a repression of the *agr* system. This allows the pathways and regulators that were repressed by the *agr* system, to become activated and increase expression of surface adhesins leading to enhanced biofilm formation and cell aggregation^57^. In a murine full-thickness burn biofilm model, genes involved in anaerobic metabolism (*ureB, ureC, arcC, acrR*, and *arcB*), adhesion (*sasF* and *sdrC*) and virulence (*luks-PV, hla*, and *splF*) were induced in a mixed-species biofilm^27^. The induction of the leukocidin, *luks-PV*, confirms previous work showing that leukocidins, specifically the Panton–Valentine leukocidin (PVL) and HlgAB enable *S. aureus* biofilm persistence within a burn wound. These leukocidins are capable of triggering NET-associated neutrophil death, preventing neutrophil-mediated biofilm clearing. They are secreted in a biofilm specific manner and their activity is independent of neutrophil-biofilm contact^58^. Like *P. aeruginosa*, a greater understanding of the role of biofilm formation in the recalcitrance of *S. aureus* wound infections is required to help mitigate their clinical burden.

### *A. baumannii* biofilm in burn wound infection

*A. baumannii* is an aerobic opportunistic Gram-negative coccobacillus. This pathogen tends to target areas of skin that are exposed through accident or injury^59^. *A. baumannii* has been a major cause of serious infections among soldiers since its proliferation among the US military treatment facilities during the Iraq–Afghanistan wars^60^. Globally about 45% of all *A. baumannii* isolates are multidrug-resistant and in Latin America and the Middle East this rate increases up to 70%^61^. Thus, one of the main global public health challenges is the dissemination of MDR *A. baumannii,* which can cause localized outbreaks particularly within burn and intensive care units^62,63^. *A. baumannii* has a remarkable capacity to survive and spread in the hospital environment due to its ability to survive on both biotic and abiotic surfaces under desiccated conditions^64^. Like *S. aureus* and *P. aeruginosa*, QS plays a major role in regulating biofilm formation in *A. baumannii* and is mediated by an AHL-based system, AbaI/AbaR^65^. In *A. baumannii,* the AbaR receptor protein forms a complex with the AbaI (auto-inducer synthase)-generated *N*-(3-hydroxydodecanoyl)-l-homoserine lactone and influences biofilm formation and surface motility. MDR *A. baumannii* strains isolated from burn patients have demonstrated high levels of *abaI* expression, leading to increased production of biofilm-associated factors such as the extracellular polysaccharide poly-b-1,6-*N*-acetylglucosamine (PNAG)^66,67^. The initial step for *A. baumannii* colonization and subsequent host infection is mediated by *csuA/BABCDE* operon encoding for pili production. The *csuE* gene facilitates the tip adhesion and its inactivation results in the abolition of pili production and biofilm formation^68^. Accordingly, *csuE* was shown to be highly expressed in strong biofilm-forming strains isolated from burn wounds. This suggests that the expression of *csuE* is an important factor controlling biofilm formation in burn wounds^67^. Relative to the other priority pathogens, comparatively little is known about the molecular mechanisms that govern *A. baumannii* in vivo biofilm formation, however, given the emergent threat posed by MDR strains of *A. baumannii*, this is likely to be an area of considerable research focus in the coming years.

### Polymicrobial biofilms

There is a growing understanding that opportunistic pathogens are rarely found in isolation in the wound microenvironment. Indeed, it has been demonstrated that there can be a diverse microbiome colonizing a healing wound, largely composed of local skin commensals, with Gram positives being the first to colonize, due to their increased capacity to withstand thermal insult subsequently followed by Gram-negatives. The composition and diversity of this wound microbiome are thought to have a significant impact on wound healing dynamics and specific commensals such as *Propionibacterium* have been linked to reduced risk of infection^69,70^. When the wound becomes infected with a known pathogen, the species diversity drops dramatically and the pathogen becomes the dominant strain recovered. Polymicrobial infections occur in up to 57% of wounds^71^. The clinical relevance of polymicrobial biofilms in wounds is still debated. While many different pathogenic species can be co-isolated from infected wounds, this is only suggestive of a polymicrobial biofilm and spatial/nutrient restrictions within the wound itself may mean that these pathogens exist as multiple monospecies biofilms within the same wound^26^. However, the impact of these biofilm-associated multispecies infections on wound healing is clear, as they are more pathogenic and delay wound healing compared to monospecies biofilms^15,72^. This is due to mutualistic, antagonistic, and synergistic interactions between pathogens occurring within the wound to drive pathogenesis and invasion^15,73^. Co-infection between the priority burn wound pathogens is common, with *S. aureus* frequently being co-isolated with *P. aeruginosa* from infected burn wounds^74–76^. Despite this, co-culturing these pathogens in vitro to study their interaction can be problematic with *P. aeruginosa* frequently outcompeting *S. aureus* in a QS-dependent manner^77,78^. In vitro evidence suggests that *S. aureus* can promote the attachment of *P. aeruginosa* to keratinocytes while *P. aeruginosa* has been shown to stimulate *S. aureus* tissue invasion^79^. When clinical burn wound isolates of these strains were grown in a drip flow biofilm reactor, co-culture lead to the formation of a more layered biofilm structure compared to monoculture^71^. Co-infection has also been shown to increase the capacity of these pathogens to survive antibiotic treatment and stimulate virulence factor production including PVL and α-hemolysin^78,80,81^. However, these impacts have been shown to be dependent on the oxygen availability and nutrient profile of the media the bacteria are cultured in. It is likely that the chemical composition of BWE could impact these synergistic phenotypes also. The host response is also impacted in a *S. aureus–P. aeruginosa* biofilm with elevated levels of the inflammatory cytokines IL-1β and TNF-α, and delayed wound re-epithelialization due to suppression of growth factor KGF1^71,78^. It was also demonstrated using mutant strains that the ability of *S. aureus* to form a biofilm is required for the elevated virulence seen in polymicrobial biofilms^72^. Co-infection of burns with *P. aeruginosa* and *A. baumannii* has been shown to have a marginal impact on wound closure but barrier skin function is significantly compromised. This was determined by quantifying trans-epidermal water loss^51^. Interestingly, it has also been shown that *A. baumannii* and *P. aeruginosa* can sense and respond to each other’s quorum-sensing molecules and increase levels of biofilm formation as a result of this signaling promiscuity^82^. This microbial crosstalk may be responsible for the exacerbated impact of these polymicrobial biofilms on the host.

### Research models of burn wound biofilms

In vitro and ex vivo models of burn wound trauma and infection are in high demand, as they enable higher-throughput studies with minimal ethical concerns. In addition, they are typically less financially challenging. A number of in vitro burn wound models have been established. Examples include the murine fibroblast model, where a heated cell culture plate is used to induce thermal injury and the fully reconstructed epidermis model which uses fibroblast-populated rat collagen gels^83–85^. Ex vivo burn models have gained popularity in the last couple of years also, due to the preference of topical burn wound treatments over systemic treatments. Skin explants are used for studying the inflammation and repair mechanisms, as well as validating treatment strategies. These ex vivo models are versatile and allow for the recapitulation of various burn types. They also provide a 3D structure for studying intercellular interactions, which are crucial in the case of a biofilm-associated infection. The porcine ex vivo burn wound model, in particular, has been used to test biofilm formation and treatments extensively^86^. It has provided insights into the antibiofilm and antibacterial activity of a hydrogen sulfide releasing peptide hydrogel on *S. aureus* biofilms^87^. However, human skin explants can only be obtained as a result of complex surgery and under The Code of Ethics of the World Medical Association. A major limitation to explant models also is that the tissue can only be viably maintained for a short period of time, due to the lack of blood and nutrient supply in vitro^88,89^. While the complex procedure of obtaining a skin explant is solvable by the wide distribution of the porcine ex vivo skin model, the challenge of the short lifespan of the explants is still an issue^86^.

### Murine model

Burn wounds present in various shapes and sizes and rodent models are versatile enough to study the majority of them. Mice and rats are considered loose-skinned animals as their skin lacks strong adherence to the underlying structure in comparison to human skin, nevertheless, they are a major contributor to burn research^90,91^. It is possible to recreate a thermal wounds by fire, hot liquid, chemicalburns, and radiation burns of any degree in rodents^92–95^. Rodents are also an adaptable model to study a variety of human burn wound pathogens including Gram-positive and -negative, anaerobic and aerobic bacteria and fungi^95–98^. This versatility encourages scientists to study biofilm infections in vivo using murine models^28,99^. The burn is usually carried out on a large skin area with manually reduced hair coverage, and animals are anesthetized prior to the procedure and offered fluid resuscitation^100,101^. Infection is established by the topical application of the culture or by injection under the burn surface^102,103^. Once the infection is established, biofilm formation can be determined by quantifying bacterial tissue load (~1 × 10^9^ cfu/g indicative of robust biofilm formation) and the expression of biofilm-associated genes^27,104^. The rat Walker-Mason scald burn and surface infection model has been an integral tool in developing our understanding of the invasive nature of burn wound infection and burn wound biofilms. This model involves the exposed back of the animal being placed in near-boiling water for 3 s for partial-thickness burn wounds or 6 s for full-thickness burns followed by the topical application of bacteria to the wound. The model has also played a major role in the clinical development of protective burn wound dressings. It has been modified and refined since its initial use in 1960s and a recent adaption has been used to study polymicrobial biofilms further demonstrating that biofilm formation contributes to burn depth progression and an increase in circulating innate immune cells^27^. Prior to this study the formation of polymicrobial biofilms in burn wounds in vivo typically required the transfer of foreign material pre-seeded with a biofilm to the burn wound site^105^.

Similar to other animal models, rodent models have their limitations. Wound healing happens by contraction in contrast to re-epithelialization and granulation seen in humans^106^. This reduces the healing time and often means that rodents are less prone to sepsis and burn wound related immunosuppression. This can create difficulties when studying chronic wounds or wound biofilm formation, however these can be overcome by artificially keeping the wound open^107^. The environmental microbiome is an important consideration in murine models, even those reared in pathogen-free environments are naturally exposed to fecal bacteria, meaning contamination is likely to occur, which presents a challenge for studying infection and biofilms using this model^91^. Given the impact of nutrient availability on biofilm formation, an important factor to consider is the macromolecular composition of BWE and how it can differ between different murine models and between that of human BWE as this can influence experimental outcomes. Regardless of their limitations, murine models remain a major contributor to expanding the understanding of burn wound biofilm infections.

### Porcine model

The porcine model has been used in medical research since Ancient Greece and over the course of time it has developed into one of the most promising xenotransplantation donor models for burn wound patients^108^. Therefore, the similarity between porcine and human physiology and anatomy has long been established^109^. The Wound Healing Society also recommends the use of pig as the primary preclinical model for wound studies^110^. Porcine skin is similar to humans in its architecture—the dermis and epidermis are thick and are similar in depth to humans and the skin is attached tightly to the underlying structures^108^. The animal hair cover is dense in restricted regions and sparse on the rest of the body. In addition, the density and distribution of blood vessels is similar to humans, as are epidermal enzyme patterns and tissue turnover time^111,112^. The healing process in pigs occurs through inflammation, proliferation, re-epithelization, and remodeling, analogous to humans. The wound creation procedure typically involves the area first being shaved and sterilized with a wash. A metal device (Brass, stainless rod) is heated to ≥100 °C and applied to the skin for a defined time period depending on the burn depth required. Bacteria are then topically applied to the wound to establish infection^51,113^. The porcine partial- or full-thickness burn wound model has been widely used for studying biofilm formation in a range of species including *S. aureus, P. aeruginosa*, *A. baumannii*, *Bacillus subtilis*, and *Enterococcus faecalis*. The porcine model has also been adapted to study mixed-species biofilms with a mixed inoculum being rubbed into the wound sufficient to establish a polymicrobial biofilm^51,114,115^. The porcine burn wound biofilm model has provided some crucial data in the development of experimental burn dressings and treatments, such as developing phage therapies for *A. baumannii* and *P. aeruginosa* biofilms in burns. It has also provided an insight into the impact of *S. aureus* biofilm formation on regeneration as a result of reduced collagen production^114,116–119^. Compared to other animal models, the porcine skin has been used extensively for ex vivo studies of burn injury and infection, avoiding the complications of in vivo models^86,120–122^. Despite all the aforementioned advantages it is important to consider that the burn trauma and concomitant infection procedure is an extremely intrusive and distressing procedure for the animal. The porcine model is also a costly and extremely high maintenance model. In addition, the size of the animal puts them at a higher risk of developing an unsolicited wound infection, meaning that a greater care is required when handling this model.

### Emerging models: *Galleria mellonella*

**Fig. 2:**
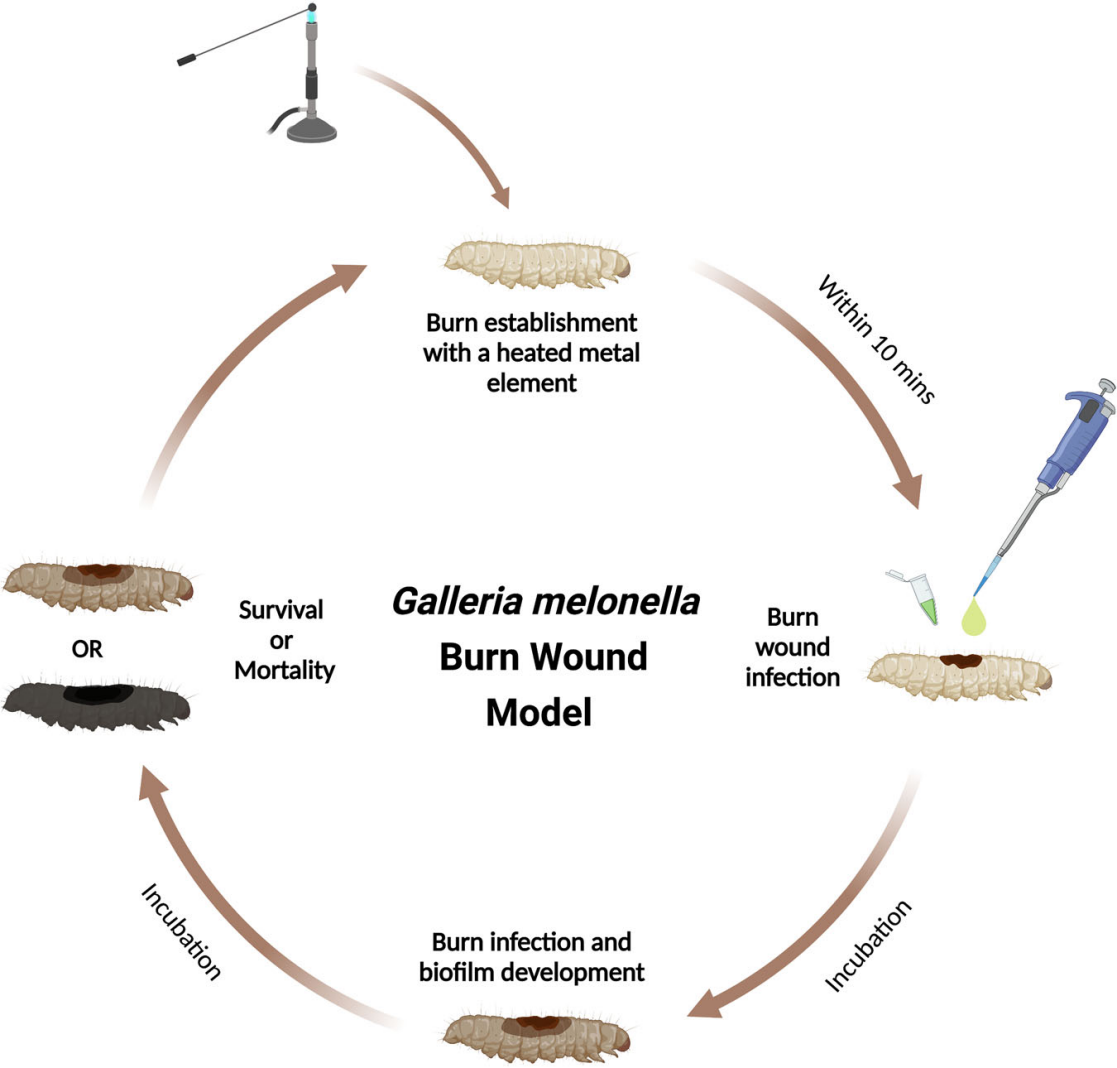
The *G. mellonella* burn wound and infection model. The burn is established on the back of the larvae with a heated metal element, closely followed by inoculation of the wound with pathogens. The infection is then allowed to establish and the biofilm to develop during the incubation process. The survival and mortality rates can be easily recorded throughout the incubation steps due to the melanisation of the larvae which is proportional to the severity of their condition. Additional wounds can be established by repeating the procedure as described. Created with BioRender.com.

*Galleria mellonella*, the greater wax moth larva, has emerged as a robust model to study microbial pathogenesis over the last decade, being used in a variety of drug toxicity, virulence and genetic mutant library screening assays. It has also been developed to study biofilm formation on implants^123^. Various methods have emerged to study Gram-positive and -negative bacterial and fungal biofilm formation in this invertebrate^123,124^. Recently a *G. mellonella* burn wound model was developed. Wound healing in this invertebrate has been shown to occur through re-epithelialization as seen in humans^125^. This model has been shown to exhibit many of the hallmarks of burn wound trauma and infection seen in mammalian models such as the correlation between the size of the wound and survival prognosis, the survival benefits of rehydration therapy and the drastic increase in mortality after the topical burn wound infection. Prior to the wound procedure, the proposed wound site is cleaned and sterilized using an ethanol wash. Like the porcine and murine models, burn wounds can then be induced by heating a metal instrument (Stainless steel) to >100 °C and applying the device to the back of the larvae for ~4 s. Infection can then be established by the topical application of bacteria to the wound site (Fig. 2). This model has been tested with *P. aeruginosa, S. aureus,* and *A. baumannii*. Each of these pathogens decreased the survival rates of infected larvae. This model can also be used to study biofilm formation at the burn wound site and determine the pathogenicity of high biofilm-forming strains^126^. Compared to the higher Eukaryotic model, there are a number of limitations with this model, one of which is the lack of an adaptive immune system. However, *G. mellonella’s* innate immune system is similar to that of mammals and this has allowed it to gain an increasing popularity in a range of research fields^127^. The innate immunity of wax moth larvae is divided into cellular and humoral responses. The role of blood is performed by hemolymph. It contains hemocytes, which are involved in phagocytosis, encapsulation of pathogens and clotting. The humoral response consists of antimicrobial peptides, lytic enzymes, opsonins, and melanization. The latter plays an important role in wound healing and sclerotization^128^. Another limitation is that the skin is different in structure to that of humans with a single epidermal layer covered by an endocuticle and an exocuticle layer^129–131^. Compared to other models, there is limited production of BWE from a larval wound.

There are however, significant advantages to this model compared to the previously described in vivo models which make it an attractive model for research. It is low cost, relatively low-maintenance, and less ethically challenging as larvae are not included in the Animal Act 1986. Critically, the *G. mellonella* model also overcomes the key limitation of scale compared to other models, allowing for large cohort studies and screening strategies that are not possible with traditional mammalian burn models. This capacity could allow this model to unclog drug discovery bottle necks and act to reduce, reuse and refine the numbers of higher eukaryotes used in burn wound studies.

### Other models

A range of other in vivo models are available although their use in biofilm-associated infection studies is comparatively limited. The rabbit ear model is widely used for wound healing research. The cartilage tissue in the ear heals through re-epithelialization and granulation instead of contraction, thus providing a better example to study hypertrophic scar formation than rats. The vascular density of this region is also very high, similarly to the vascularization of human dermis^132^. The rabbit ear model has been used to study monospecies and polymicrobial burn wound biofilms and its use in burn wound research has led to a number of useful insights, such as enhancement of *P. aeruginosa* infected wound healing by negative pressure^72,133^. Canine models have been used to study burn wound healing and to test therapeutic agents^134^. This model faces major ethical complications, it is not cost-efficient, and it has loose skin around the main body. This model has been used to establish antimicrobial and regeneration-promoting properties of kefir in third-degree burn wounds^135^. Ovine models have also been used to study burn wound healing. As with any other large animal model, the sheep experimental cohort is limited to very few animals. However, this model has been used to study flame burns, smoke inhalation injury, wound repair, and regeneration and therapeutic lead testing^136–139^.

## Conclusions

**Fig. 3:**
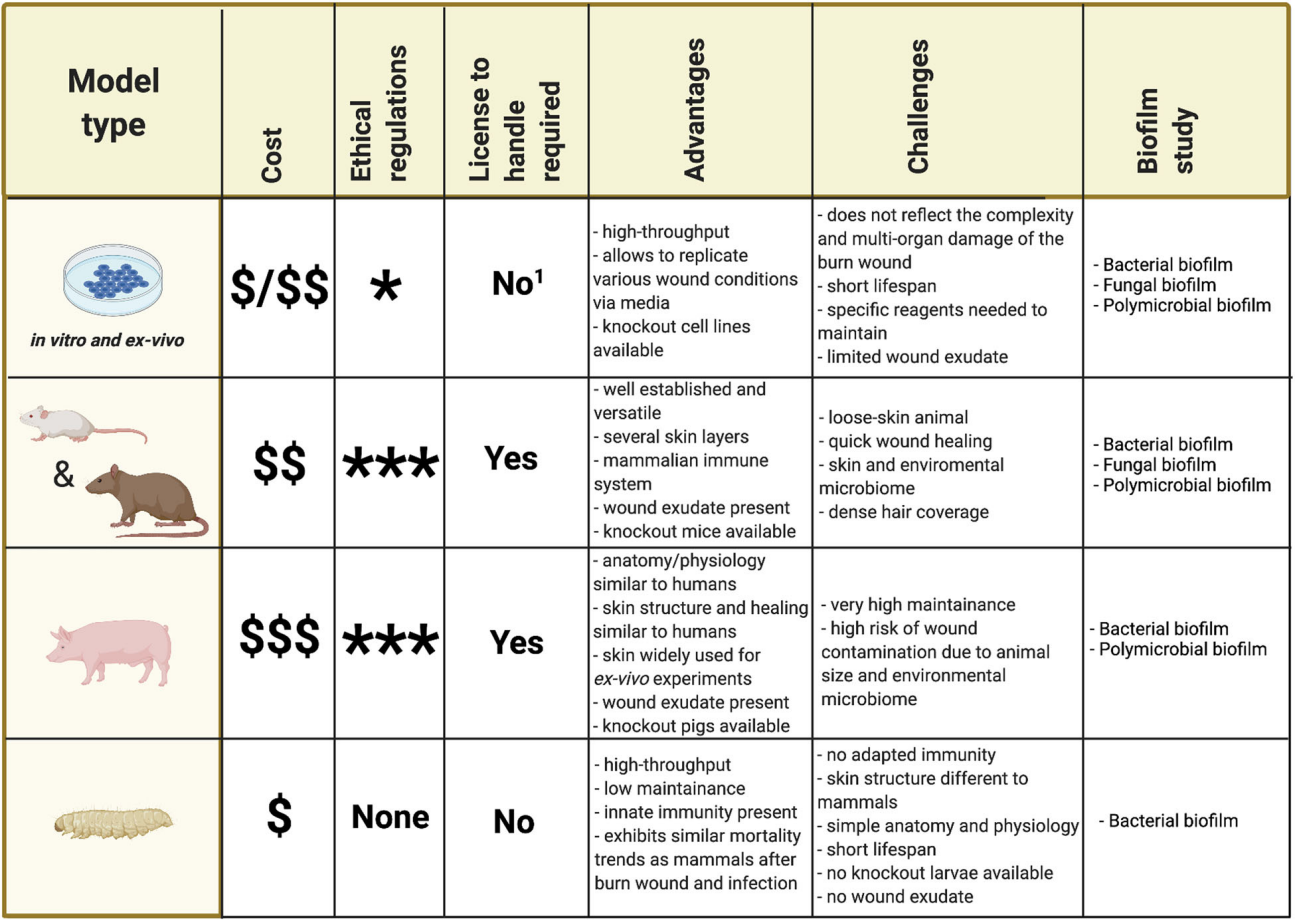
A comparative table of the burn wound and infection models. ^1^Licensing is required on a case-by-case basis for human explants. Created with BioRender.com.

Given the enormous health and financial burdens of infection in burn wound care, understanding the role of biofilm formation in burn wound pathogenesis and delayed wound healing is a major research priority. The models outlined in this review (Fig. 3) will help answer pivotal questions facing the research field such as understanding the distinction between transient colonization and infection and the role of biofilm formation in acute infection. The emerging role of the wound microbiome and the impact that it has on wound healing and limiting infection progression will also be better understood using these models. With all the valuable insights that in vitro and ex vivo models are providing, the need for in vivo models remains. Each of the in vivo models outlined in this review have differing advantages and challenges which should be considered in the context of the biological question being asked to reduce and refine the use of live animals in burn wound research and to maximize the scientific outputs.
